# Disordered leptin and ghrelin bioactivity in adolescent idiopathic scoliosis (AIS): a systematic review and meta-analysis

**DOI:** 10.1186/s13018-020-01988-w

**Published:** 2020-10-30

**Authors:** Qi Wang, Chi Wang, Wenhao Hu, Fanqi Hu, Weibo Liu, Xuesong Zhang

**Affiliations:** 1 Medical School of Chinese PLA General Hospital, Beijing, 100853 China; 2 Department of Orthopedics, the First Medical Center, Chinese PLA General Hospital, 28 Fuxing Road, Haidian District, Beijing, 100853 China; 3 Department of Clinical Laboratory Medicine, the First Medical Center, Chinese PLA General Hospital, Beijing, 100853 China

**Keywords:** Adolescent idiopathic scoliosis, Leptin, Ghrelin, Soluble leptin receptor, Meta-analysis

## Abstract

**Background:**

Adolescents with scoliosis consistently demonstrate lower body weight, lean muscle mass, and bone mineral density than healthy adolescent counterparts. Recent studies have focused on understanding how leptin and ghrelin signaling may play a role in adolescent idiopathic scoliosis (AIS). In our current study, we aim to evaluate the serum levels of leptin, soluble leptin receptor (sOB-R), and ghrelin in AIS patients through systematic review and meta-analysis.

**Methods:**

We conducted our systematic review by searching the keywords in online databases including PubMed, Embase, Cochrane, Elsevier, Springer, and Web of Science from the time of database inception to January 2020. Inclusion criteria were studies that measure leptin, soluble leptin receptor (sOB-R), and ghrelin levels in AIS patients. Selection of studies, assessment of study quality, and data extraction were performed by two reviewers independently. Then, data was analyzed to calculate the mean difference and 95% confidence interval (CI).

**Results:**

Seven studies concerning leptin/sOB-R and three studies concerning ghrelin were qualified for meta-analysis (one study concerning both leptin and ghrelin). Serum leptin of patients with AIS were significantly lower when compared with healthy controls, with the weighted mean difference (WMD) of − 0.95 (95% CI − 1.43 to − 0.48, *p* < 0.0001) after reducing the heterogeneity using six studies for meta-analysis, while sOB-R and ghrelin level was significantly higher in AIS group when compared with control group, with the WMD of 2.64 (95% CI 1.60 to 3.67, *p* < 0.001) and 1.42 (95% CI 0.48 to 2.35, *p* = 0.003), respectively.

**Conclusion:**

Our current meta-analysis showed that serum level of leptin in AIS patients was significantly lower when compared with control subjects, while serum sOB-R and ghrelin levels were significantly higher in AIS patients. More clinical studies are still required to further validate the predictive value of leptin or ghrelin for the curve progression for AIS patients.

## Introduction

Adolescent idiopathic scoliosis (AIS) is a complex tridimensional deformity, characterized by rotation of the vertebrae and lateral deviation of the spine. AIS occurs in adolescents between 10 and 18 years old and affects approximately 5% of all children and mostly girls, with a ratio of 10 girls to each boy [1]. What makes AIS a tough clinical problem is that 28% patients could still demonstrate curve progression even after bracing, thus making surgery the only way to treat severe curves [2]. Currently, there is no widely agreed etiology of AIS. Several theories on the pathology of AIS strongly indicate that AIS is a multifactorial disease that involves genetic factors, hormones and neuromuscular, environmental, and lifestyle factors [3]. In addition, several studies had found that AIS patients have common features of taller stature, lower body mass index (BMI), and systemic low bone mass [4]. It is also accepted that AIS is a systemic disease, and scoliosis mainly results from the abnormal systemic skeletal growth and the asynchronous spinal neuro-osseous growth [5].

Recent work has focused on understanding how leptin and ghrelin signaling may play a role in producing the altered BMI in AIS [6, 7]. Leptin is coded by the leptin gene (Ob) and is primarily expressed in white adipose tissue. It binds to leptin receptor (LEPR) and plays key roles in modulating bone formation by regulating the expression of several neuropeptides in the hypothalamus and inducing sympathetic activation [8]. Bone marrow stromal cells are reported to be directed to osteogenesis by leptin, instead of adipogenic pathway [9]. Soluble leptin receptor (sOB-R) serves as a mediator of leptin’s action as it can delay the clearance of leptin by binding leptin in the bloodstream. Therefore, sOB-R might affect the leptin bioavailability in the circulation through competing with the membrane-anchored LEPR and inhibiting the transport of free leptin into cells and across the blood-brain barrier [10]. Previous studies have observed an abnormal leptin level in skeletal growth and its signaling pathway for the etiology of AIS. In a study conducted by Qiu et al. [11], serum leptin levels were found to be significantly lower in AIS, while Liu et al. [12] claimed that the serum total leptin level between AIS and healthy girls is similar after adjusting the BMI. In addition, previous studies had reported higher sOB-R level in patients with AIS, and genome-wide association study had also identified genetic determinants of plasma sOB-R levels in LEPR gene [13].

Ghrelin is a type of polypeptide hormone mainly from the stomach and also the first endogenous ligand of growth hormone secretagogue receptor-1A (GHSR-1A) [14]. It is reported to regulate growth hormone secretion, energy metabolism, sleep, gastrointestinal function, and bone formation. Recent findings suggest that ghrelin is correlated with the abnormal development of cartilage in patients with scoliosis, which may affect the development of spinal deformities [15]. Currently, several studies concern the changes of ghrelin in AIS, and we aim to perform a meta-analysis based on these publicized papers.

Therefore, in our study, we aim to evaluate papers concerning the serum levels of leptin, sOB-R, and ghrelin in AIS patients to summarize the available evidences on the correlation between abnormal leptin or ghrelin levels with the risk of AIS.

## Methods

Data from the selected studies were extracted, and eligible studies were assessed by means of the revised Quality Assessment of Diagnostic Accuracy Studies (QUADAS-2) criteria [16]. Statistical analysis, evidence synthesis, and report compilation were carried out as the steps below. We strictly adhered to standards of the Preferred Reporting Items for Systematic Reviews and Meta-Analyses (PRISMA) in reporting the findings of this review (Supplemental Table1 for PRISMA detailed checklist).

### Search strategy

We searched the electronic databases including PubMed, Embase, Web of Science, the Cochrane Library, and Science Direct for entries recorded from the time of database inception to February 2020. We used keywords or mesh words as follows: “adolescent idiopathic scoliosis” to represent the disease, “leptin” or “soluble leptin receptor” or “sOB-R” or “ghrelin” as our target index.

### Study selection

**Fig. 1:**
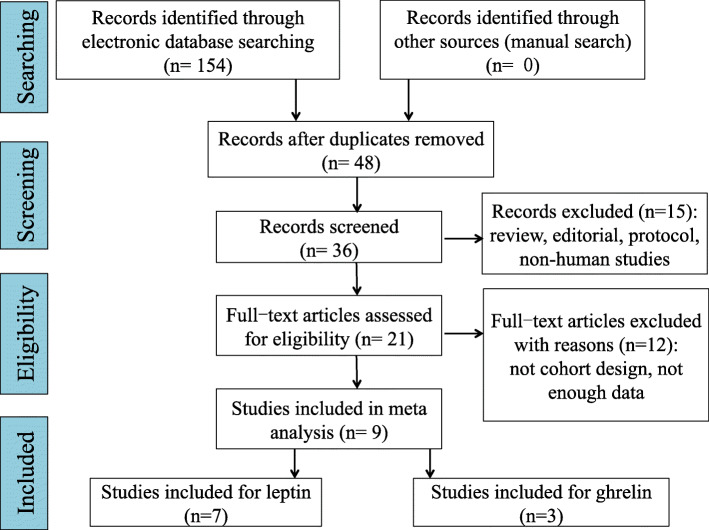
Screening of articles. PRISMA flow diagram for study selection showing the number of studies identified, screened, eligible, and included in the systematic review and meta-analysis

Screening was performed as follows. Two researchers independently reviewed the title and abstract of each assay to select papers, which require full-text screening. In the initial stage of the screening, 10 articles should be used to confirm the agreement between the researchers. When confronted with disagreements, two researchers had to come to a consensus about the screening standard. After full-text screening, a list of reasons for exclusion was performed. Inclusion criteria were as follows: (1) randomized-controlled trials or high-quality observational studies; (2) studies that compared serum leptin, sOB-R, or ghrelin of the patients who were diagnosed as AIS; (3) sufficient data could be extracted for meta-analysis. Exclusion criteria were as follows: (1) non-clinical studies such as basic science studies, narrative reviews, surveys, letters, editorials, case series, case reports, comments, conference abstracts, or expert opinions; (2) non-English studies. The flow diagram is illustrated in Fig. 1.

### Quality assessment

The methodological quality of the included studies was appraised by an adapted version of the QUADAS-2, which is composed of four key domains (patient selection, index test, reference standard, and flow and timing). Signaling questions were applied to evaluate the risk of bias and clinical applicability. These questions were responded as “yes” for low risk of bias/concerns, “no” for high risk of bias/concerns or “unclear”.

### Data extraction

The following detailed information of qualified studies was extracted: (i) study characteristics including author, year of publication, country, and sample size; (ii) population characteristics including patients’ mean age, sex, body mass index (BMI), and method of assessment; (iii) outcomes of tested proteins including serum leptin, sOB-R, and ghrelin.

### Statistical analysis and heterogeneity assessment

Meta-analysis was conducted with Review Manager (version 5.3) using the eligible studies. An *I*^*2*^ was used to assess heterogeneity. For meta-analysis, in which the *I*^2^ value was under 40%, the fixed model was used. For meta-analysis, in which the *I*^2^ value was higher than 40%, a random model was used. For all effect estimates, a value of *p* < 0.05 was considered to be statistically significant. The outcomes were estimated as pooled mean and 95% confidence intervals (CI).

## Results

### Research results

**Fig. 2:**
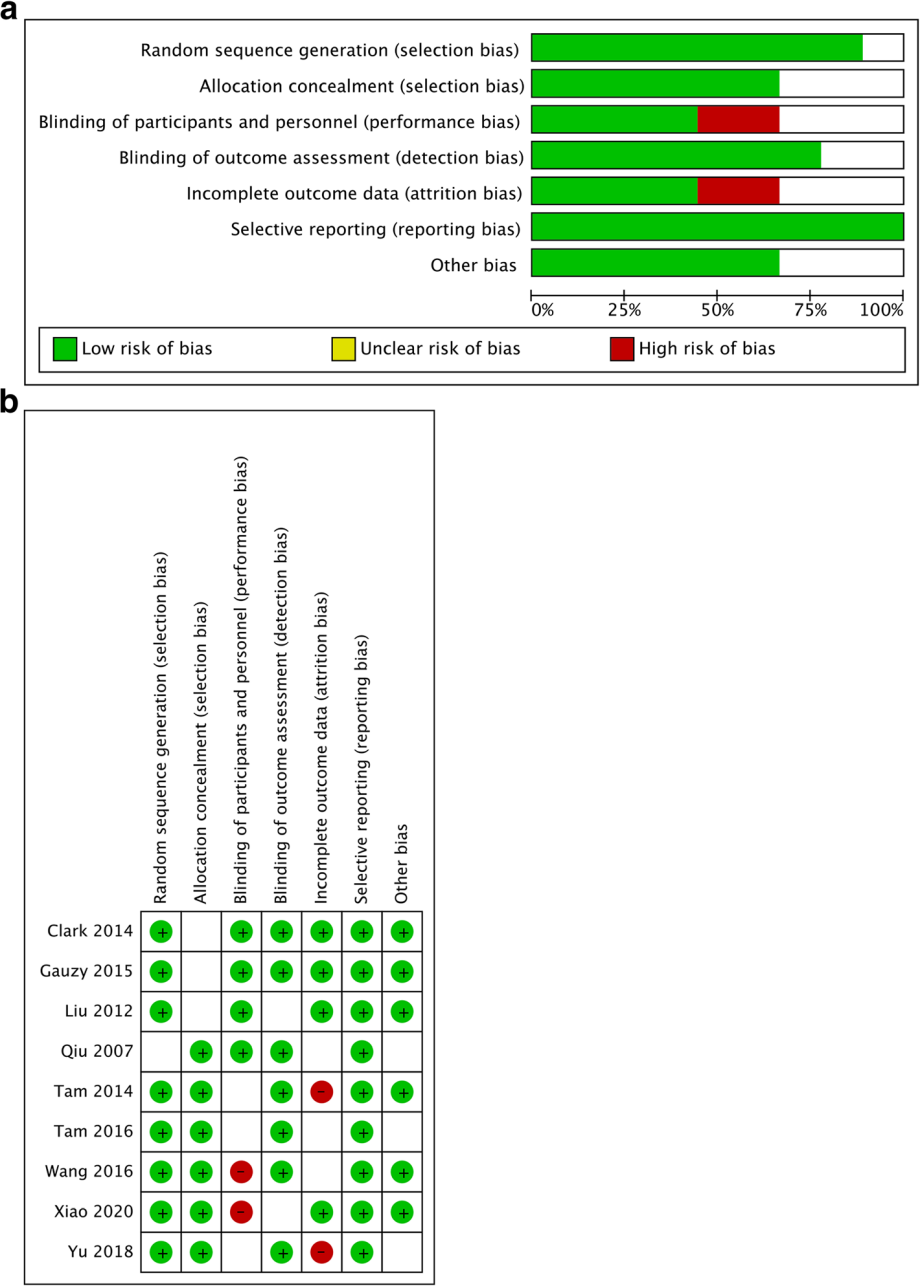
Quality assessment of included studies using QUADAS-2 tool criteria

The PRISMA statement flowchart shows the process of literature screening, study selection, and reasons for exclusion (Fig. 1). Of the identified initial 154 articles, 48 of them were left for further screening after excluding the duplicates. Another 12 articles were excluded after reading the title and abstract, reasons including inappropriate article type (reviews, comments, letters, or non-human studies). Then, the remaining 36 articles were read through, and 25 were unqualified due to inappropriate study or incomplete data for systematic review. Eventually, 9 studies were qualified for this meta-analysis [11, 12, 17–22]. QUADAS-2 quality assessment for the included studies is shown in Fig. 2.

### General characteristics of studies

The extracted information from each paper, including first author, year of publication, number of cases and controls, and mean levels of leptin and ghrelin, is shown in Table 1. Among these included 9 articles, 6 articles explored changes of serum leptin and sOB-R in AIS [11, 12, 17–19], another 2 explored changes of ghrelin in AIS [21, 22], while the remaining one study both leptin and ghrelin in AIS [20]. A total of 700 AIS patients and 3536 healthy controls were included for the meta-analysis of leptin, while 198 AIS patients and 138 healthy controls were included for ghrelin analysis. Of the included studies, five had been published in the last 5 years, and eight in the last 10 years; only one was published 13 years ago. Two studies were conducted in European countries (UK or France), while the remaining seven studies were all conducted in China (Nanjing, Hong Kong, and Changsha). Among the included studies, the sample size varied from 45 to 3332, and all studies use teenage girls for AIS and healthy control groups.

**Table 1:** General information of included studies

Author (year)	Country	Age (year) (AIS/control)	BMI (kg/m^2^) (AIS/control)	No of case (AIS/control)	Leptin (ng/ml) (AIS/Control)	sOB-R (ng/ml) (AIS/Control)	Free leptin index (Leptin/sOB-R) (AIS/Control)	Ghrelin (pg/ml) (AIS/control)	Detection method
Qiu et al. 2007 [11]	China	13.5/15.1	17.5 ± 0.2/20.9 ± 0.4	47/80	8.6 ± 0.8/14.9 ± 0.9				ELISA
Liu et al. 2012 [12]	China	13.7 ± 1.5/13.8 ± 1.4	17.8 ± 2.3/19.5 ± 2.2	95/46	7.0 ± 5.0/8.4 ± 5.8	23.7 ± 6.7/20.5 ± 5.6	0.33 ± 0.25/0.48 ± 0.41		ELISA
Clark 2014 [23]	UK	None	17.2 ± 2.9/17.6 ± 2.8	180/3152	5.24 ± 4.26/6.28 ± 5.11				ELISA
Tam et al. 2014 [18]	China	13.05 ± 0.52/12.93 ± 0.43	17.91 ± 2.20/18.64 ± 2.15	94/87	6.75 ± 5.01/7.26 ± 4.99	25.86 ± 6.49/23.43 ± 5.27			ELISA
Tam et al. 2016 [19]	China	12.91 ± 0.63/12.96 ± 0.45	17.59 ± 2.10/18.35 ± 2.23	148/116	8.1 ± 7.48/10.47 ± 8.34	26.46 ± 6.66/23.98 ± 6.95	0.28/0.39		ELISA
Wang et al. 2016 [17]	China	12.81 ± 1.82/13.67 ± 1.67	17.66 ± 1.15/19.66 ± 0.91	31/15	7.62 ± 2.80/8.89 ± 4.15				ELISA
Yu et al. 2018 [20]	China	12.43 ± 1.90/12.80 ± 1.22	17.83 ± 1.18/17.55 ± 1.35	105/40	6.55 ± 2.88/8.01 ± 3.12			6550 ± 2460/4460 ± 2020	ELISA
Gauzy et al. 2015 [22]	France	14.3 ± 1.4/13.9 ± 1.6	19.1 ± 2.6/18.5 ± 2.5	49/15				261.9 ± 120.3/146.1 ± 59.2	Radioimmunoassay
Xiao et al. 2020 [21]	China	14.52 ± 2.24/14.23 ± 2.04	17.76 ± 2.60/20.87 ± 4.54	83/44				27.33 ± 6.57/15.81 ± 3.82	ELISA

*ELISA* enzyme-linked immunosorbent assay

### Changes of leptin, sOB-R, and ghrelin in AIS girls

**Fig. 3:**
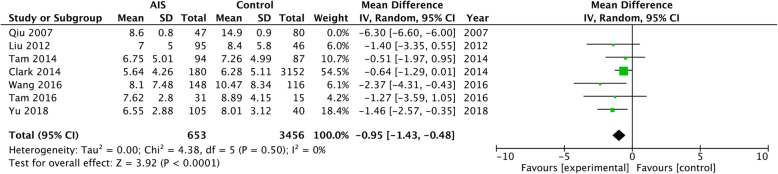
Forest plot of the meta-analysis of leptin levels in AIS and healthy controls

**Fig. 4:**
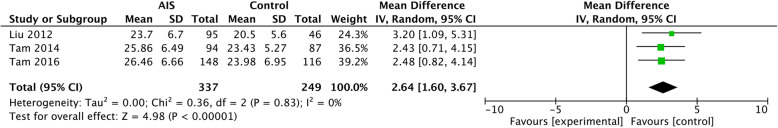
Forest plot of the meta-analysis of s-OB-R levels in AIS and healthy controls

**Fig. 5:**
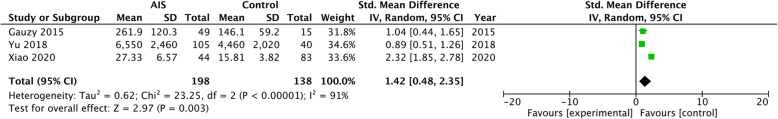
Forest plot of the meta-analysis of ghrelin levels in AIS and healthy controls

As for leptin, we performed the meta-analysis using the random-effects model. With seven included studies, the summarized result indicated no significant difference between AIS patients and control group (WMD = − 2.02, 95% CI − 4.71 to 0.67, *p* = 0.14) and also high heterogeneity (*I*^2^ = 98%, *p* < 0.0001) (Supplementary Figure 1). Then, we excluded the study conducted by Qiu et al. and found that serum leptin of patients with AIS was significantly lower when compared with healthy controls (WMD = − 0.95, 95% CI − 1.43 to − 0.48, *p* < 0.0001), and the six sets of results showed no heterogeneity (*p* = 0.50, *I*^2^ = 0) (Fig. 3). Serum sOB-R level was significantly higher in AIS group when compared with control group (WMD = 2.64, 95% CI 1.60 to 3.67, *p* < 0.001), with the heterogeneity of 0% (Fig. 4). However, patients with AIS had higher serum ghrelin than healthy controls (WMD = 1.42, 95% CI 0.48 to 2.35, *p* = 0.003), and the heterogeneity of the included three studies also was statistically significant (*p* < 0.0001, *I*^2^ = 91%) (Fig. 5).

## Discussion

Our meta-analysis suggests that leptin level in serum is significantly lower in AIS patients when compared with healthy controls, which was in accordance with the conclusion of most papers enrolled in our meta-analysis. According to the inclusion and exclusion criteria, seven studies were initially enrolled in the meta-analysis for serum leptin. Then, we firstly summarized results of seven studies but found that the heterogeneity was quite high among these studies. We wonder if the number of population influenced the results, and then, we excluded the study conducted by Clark, who included 3352 subjects in total. The heterogeneity did not change much (*I*^2^ = 97%, Supplementary Figure 2), which indicated that this study was not the most heterogeneous one. Then, we excluded the study conducted by Qiu et al. and surprisingly found that the heterogeneity decreased to 0 (Fig. 3). Thus, we consider that this study contributed most to the heterogeneity of the meta-analysis. Then, we reviewed this study again and found that they divided the AIS patients into older and younger subgroup with the mean age of 12.6 and 14.9 respectively. The leptin level we previously enrolled belonged to the older AIS patients and control group. In addition, the author provided “standard error” instead of “standard deviation,” the latter of which was applied in other enrolled studies. We speculated that these factors might contribute to the heterogeneity of this study. Thus, we decided to exclude the study of Qiu et al. [11] in the final meta-analysis. Although the final result for summarized leptin level was changed, we considered that results with the lower heterogeneity were more stable and credible.

AIS patients have common features of taller stature, lower body mass index (BMI), and systemic low bone mass. These specific characteristics in AIS patients had been hypothesized to be correlated with leptin, which could act through both central and peripheral pathways to regulate osteoblastic differentiation and osteoclast activity [24]. Leptin has been reported to play an important role in regulating energy expenditure, bone metabolism, body weight, and hypothalamic-pituitary system. In addition, systemic leptin administration in animals and humans usually exerts a positive effect on bone mass [24]. For example, leptin could exert dose-related increase in the formation of mineralized bone nodules in primary osteoblast cultures, differentiation of marrow stromal cell into osteoblast phenotype, and synthesis of bone matrix proteins and osteocalcin [25]. Since the publication of the first paper concerning the correlation of leptin and AIS by Qiu et al. [11], several other researchers also found decreased level of circulating leptin in AIS patients and suggested that leptin dysfunction might play an important role in the development of AIS. Liang et al. further investigated whether the alteration of leptin level is a primary event (as a result of variations in gene) or a secondary one (as an outcome). They studied the polymorphisms in exons, untranslated regions of leptin gene, and expression of leptin and leptin receptor in AIS adipocytes and osteoblast. Their results indicated that polymorphisms of leptin gene have no association with AIS, but expression of leptin receptor is decreased in AIS, which may lead to hyposensitivity of AIS to leptin.

Then, studies began to pay attention to leptin receptor, and sOB-R is found to bind leptin in the circulation [10]. sOB-R might compete with membrane-anchored leptin receptor and inhibit the transport of free leptin into cells. Transgenic mice with sOB-R over-expression were found to have increased circulating total leptin and enhanced leptin signal [26]. The function of sOB-R has been reported to be complex; on the one hand, it could prevent the clearance of leptin and maintain the readily available leptin; on the other hand, cell and animal models suggested an inhibitory effect of sOB-R on leptin signaling [27]. Liu et al. reported that polymorphism of rs2767485 in LEPR gene is associated with the occurrence of AIS, which indicated the role of LEPR as a candidate gene for regulating sOB-R levels [28]. Thus, dysfunction of LEPR gene may induce abnormal leptin bioavailability by affecting the expressing level of sOB-R.

In our current meta-analysis, patients with AIS had higher serum levels of sOB-R than healthy controls. It was hypothesized that leptin level in AIS ought to be higher, which was contradictory to our current results (the leptin level was not statistically significant between two groups). We speculate that there might exist a defect in the feedback loop of hormonal systems, which is supposed to regulate leptin and s-OR-B mutually. In addition, we hypothesize that this dysfunction might also influence the bioactivity of leptin on the target tissue in AIS, including the differentiation of chondrocyte and osteoblast. Abnormal response of bone tissue (for example, trabecular compartment) to leptin signaling in AIS has been reported. Thus, animal study concerning the abnormal leptin bioavailability or sensitivity in AIS is essential in the future study.

Our meta-analysis also analyzes the serum levels of ghrelin and suggests that patients with AIS had higher levels of ghrelin than healthy controls. It has been reported that ghrelin is correlated with the abnormal development of cartilage in patients with scoliosis, which may affect the development of spinal deformities [15]. Since AIS patients have increased chondrocyte activity and active endochondral osteogenesis [29], and ghrelin was shown to inhibit the apoptosis of chondrocytes through protein kinase B (Akt) and nuclear factor-kappa beta (NF-κB) pathways in osteoarthritis [30], ghrelin has been suggested as part of the etiology of AIS. In addition, expression of ghrelin receptor in the primary chondrocytes of AIS patients was significantly higher than that in common patients, and ghrelin could promote the AIS primary chondrocyte proliferation and upregulate the expression of cartilage-specific genes through extracellular regulated protein kinase (ERK)/signal transducer and activator of transcription (STAT3) pathways [15]. In addition, Yu et al. reported that high ghrelin could serve as a quantitative indicator for predicting curve progression and thus helps in precise selection of treatment in AIS patients [20]. Xiao et al. observed on significant difference in four ghrelin level-related single nucleotide polymorphisms (SNPs) between the AIS osteopenia and control groups, which indicated that abnormally high ghrelin may not result from gene variations, while dysregulation of ghrelin/receptor activator of nuclear factor-kappa beta ligand (RANKL)/osteoprotegerin (OPG) pathway may lead to decreased osteogenic ability of osteoblasts and bone marrow stem cells (BMSCs), which may be related to lower bone mass in AIS osteopenia [21].

There are several limitations in the current meta-analysis. Firstly, the number of qualified studies included for meta-analysis was still limited; only 3 studies were included for ghrelin and another 3 studies for sOB-R. This might contribute to the high heterogeneity, and due to the limited number of enrolled studies, sub-group analysis could not be realized. Secondly, only one study was conducted to explore the predictive ability of curve progression in AIS patients, and this could not be sufficient to convey a convincing conclusion on overall populations. Finally, the included patients were from Asian country (mostly from China), France, and the UK. Whether the variation trends of these two hormones could be applied to patients throughout the world requires more investigation.

In conclusion, based on this meta-analysis, serum level of sOB-R and ghrelin is higher in AIS patients than healthy controls. But the concentration of leptin was not significantly different between two groups. More clinical studies are required to further validate the predictive ability of ghrelin or leptin for the progression of AIS patients. In addition, more preliminary researches concerning the relationship between the abnormal changes of leptin/ghrelin and etiology of AIS, such as the effect of leptin/ghrelin on osteoblast differentiation and cartilage development, are also warranted.

## Supplementary information

**Additional file 1:** Supplemental Table 1 PRISMA NMA Checklist of Items to Include When Reporting A Systematic Review Involving a Network Meta-analysis

**Additional file 2:** Supplementary figure 1. Forest plot of the meta-analysis of leptin levels in AIS and healthy controls (seven studies enrolled in the meta-analysis).

**Additional file 3:** Supplementary figure 2. Forest plot of the meta-analysis of leptin levels in AIS and healthy controls (excluding the study conducted by Clark).

## Data Availability

All data generated or analyzed during this study are included in this published article.

## Abbreviations

AIS: Adolescent idiopathic scoliosis
sOB-R: Soluble leptin receptor
CI: Confidence interval
WMD: Weighted mean difference
BMI: Body mass index
Ob: Leptin gene
LEPR: Leptin receptors
GHSR-1A: Growth hormone secretagogue receptor-1A
QUADAS-2: Quality Assessment of Diagnostic Accuracy Studies
PRISMA: Preferred Reporting Items for Systematic Reviews and Meta-Analyses
Akt: protein kinase B
NF-κB: Nuclear factor-kappa beta
ERK: Extracellular regulated protein kinase
STAT3: Signal transducer and activator of transcription
SNP: Single nucleotide polymorphism
OPG: Osteoprotegerin
RANKL: Receptor activator of nuclear factor-kappa beta ligand
BMSC: Bone marrow stem cell
