# Supplementary figures and images for: Disordered leptin and ghrelin bioactivity in adolescent idiopathic scoliosis (AIS): a systematic review and meta-analysis
Source: J Orthop Surg Res. 2020 Oct 30;15:502. doi: 10.1186/s13018-020-01988-w (PMC7596938; doi:10.1186/s13018-020-01988-w)

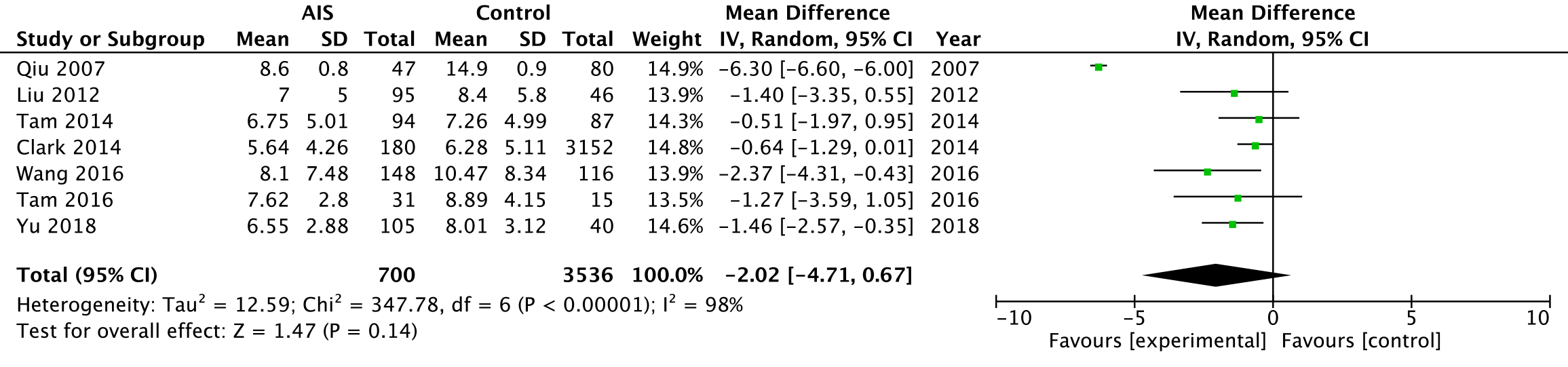

Supplement: Supplementary file 2 — Additional file 2: Supplementary figure 1. Forest plot of the meta-analysis of leptin levels in AIS and healthy controls (seven studies enrolled in the meta-analysis). [file 13018_2020_1988_MOESM2_ESM.tif]

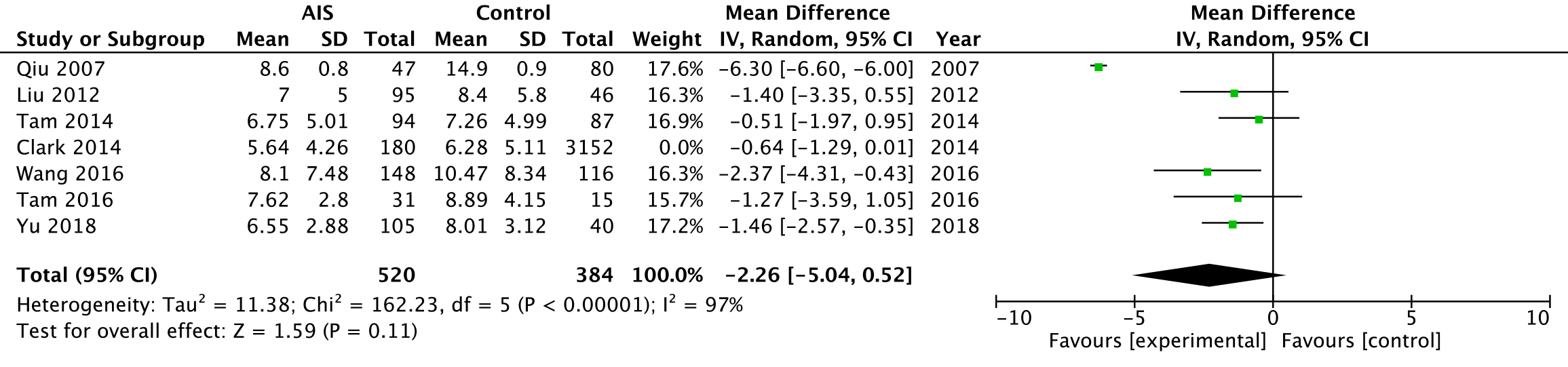

Supplement: Supplementary file 3 — Additional file 3: Supplementary figure 2. Forest plot of the meta-analysis of leptin levels in AIS and healthy controls (excluding the study conducted by Clark). [file 13018_2020_1988_MOESM3_ESM.tif]
